# Footwear, foot orthoses and strengthening exercises for the non-surgical management of hallux valgus: protocol for a randomised pilot and feasibility trial

**DOI:** 10.1186/s13047-022-00553-4

**Published:** 2022-06-03

**Authors:** Hylton B. Menz, Polly Q. Lim, Sheree E. Hurn, Karen J. Mickle, Andrew K. Buldt, Matthew P. Cotchett, Edward Roddy, Anita E. Wluka, Bircan Erbas, Shannon E. Munteanu

**Affiliations:** 1 School of Allied Health, Human Services and Sport, La Trobe University, Melbourne, VIC 3086 Australia; 2 School of Clinical Sciences, Faculty of Health, Queensland University of Technology, Kelvin Grove, QLD 4059 Australia; 3 School of Environment and Life Sciences, College of Engineering, Science and Environment, University of Newcastle, Ourimbah, NSW 2258 Australia; 4 Primary Care Centre Versus Arthritis, School of Medicine, Keele University, Keele, ST5 5BG Staffordshire UK; 5 Haywood Academic Rheumatology Centre, Midlands Partnership NHS Foundation Trust, Haywood Hospital, Burslem, ST6 7AG Staffordshire UK; 6 Department of Epidemiology and Preventive Medicine, Monash University, Alfred Hospital, Melbourne, VIC 3004 Australia; 7 School of Psychology and Public Health, College of Science, Health and Engineering, La Trobe University, Melbourne, VIC 3086 Australia

## Abstract

**Background:**

Hallux valgus is a common and disabling condition. This randomised pilot and feasibility trial aims to determine the feasibility of conducting a fully-powered parallel group randomised trial to evaluate the effectiveness of a multifaceted non-surgical intervention for reducing pain associated with hallux valgus.

**Methods:**

Twenty-eight community-dwelling women with painful hallux valgus will be randomised to receive either a multifaceted, non-surgical intervention (footwear, foot orthoses, foot exercises, advice, and self-management) or advice and self-management alone. Outcome measures will be obtained at baseline, 4, 8 and 12 weeks. The primary outcome is feasibility, which will be evaluated according to demand, acceptability, adherence, adverse events, and retention rate. Limited efficacy testing will be conducted on secondary outcome measures including foot pain (the Manchester-Oxford Foot Questionnaire), foot muscle strength (hand-held dynamometry), general health-related quality of life (the Short Form-12), use of cointerventions, and participants’ perception of overall treatment effect. Biomechanical testing will be conducted at baseline to evaluate the immediate effects of the footwear/orthotic intervention on pressure beneath the foot and on the medial aspect of the first metatarsophalangeal joint and hallux.

**Discussion:**

This study will determine the feasibility of conducting a fully-powered randomised trial of footwear, foot orthoses, foot exercises, advice and self-management for relieving pain associated with hallux valgus and provide insights into potential mechanisms of effectiveness.

**Trial registration:**

Australian and New Zealand Clinical Trial Registry (ACTRN12621000645853).

**Supplementary Information:**

The online version contains supplementary material available at 10.1186/s13047-022-00553-4.

## Background

Hallux valgus is a common and disabling condition characterised by the lateral deviation of the hallux towards the lesser toes. A systematic review of population-based studies (total 496,957 participants) reported pooled prevalence estimates of 23% in people aged 18 to 65 years and 36% in people aged over 65 years [1], with women twice as likely to be affected. The progressive subluxation of the first metatarsophalangeal joint and formation of an osseus prominence on the medial aspect of the first metatarsal leads to crowding and deformity of the lesser toes [2], abnormal gait patterns [3], impaired balance [4], difficulties with finding comfortable footwear [5], an increased risk of falls in older people [6] and decreased health-related quality of life [7].

The impact of hallux valgus on the healthcare system is considerable, as it is a frequent presentation in primary care and is one of the most common conditions treated by podiatrists [8] and orthopaedic surgeons [9]. A recently published analysis of 1.6 million patient-encounter records from the Bettering the Evaluation and Care of Health primary care dataset revealed that general practitioners (GPs) in Australia encounter an estimated 60,000 cases of hallux valgus every year (an approximate cost of AU$2.2 M in Medicare subsidies for initial GP consultations alone [10]) while Medicare Benefits Schedule data indicates that approximately 7,000 hallux valgus surgical procedures are performed by orthopaedic surgeons in the private sector per year, at an estimated cost of AU$3 million [11]. Although substantial, this is an underestimate of the true economic burden of hallux valgus surgery, as many procedures are also performed in public hospitals and by podiatric surgeons [12].

Despite the high prevalence, associated impairments in quality of life and economic burden of hallux valgus, there are no widely adopted, evidence-based clinical guidelines to inform the selection of the most appropriate interventions. In the United Kingdom, the National Institute for Health Care Excellence (NICE) [13] recommend that people with hallux valgus should first be provided with analgesia, footwear advice, bunion splints or orthoses. If these treatments are not effective or the severity of the condition progresses, surgical referral is then recommended. However, the NICE guidelines caution that these recommendations are based primarily on expert opinion rather than evidence. In Australia, where no such guidelines exist, people with painful hallux valgus presenting to GPs are most commonly provided with advice, pain medications and referral to orthopaedic surgeons [10].

Non-surgical management of hallux valgus may involve footwear advice or modification, foot orthoses, night splints, and physical therapies (manual therapy, taping or foot exercises). In podiatric clinical practice, these interventions are often combined in a multifaceted approach [14]. However, there is limited evidence for the effectiveness of any of these interventions. A recent systematic review and meta-analysis of non-surgical interventions for hallux valgus identified 16 parallel-group and crossover studies evaluating a wide range of non-surgical interventions [15]. Overall, included trials were of low methodological quality. Many had small sample sizes (12 out of 16 studies having < 60 participants) and short follow-up periods (10 out of 16 having follow-up < 3 months), thus providing low certainty as to the effectiveness of non-surgical interventions and longer-term management of the condition.

Of the available non-surgical treatments, the most widely used are footwear, foot orthoses and foot exercises [14], and there is preliminary evidence supporting the use of these approaches. Ill-fitting footwear is an important modifiable risk factor for the development and progression of hallux valgus, as it has been shown that the likelihood of having the condition is significantly greater in those who have worn shoes with a very narrow toe box between the ages of 20 and 29 years [16]. Furthermore, if the condition is already present, the provision of extra-depth footwear which accommodates the broader forefoot may reduce pain [17]. Foot orthoses have been shown to reduce load under the great toe and medial midfoot in people with hallux valgus [18], and a randomised trial reported a significant reduction in pain at six months those who received this intervention [19]. As hallux valgus progresses, the malalignment of the first metatarsophalangeal joint results in decreased muscle size [20] and subsequent weakness of the muscles responsible for hallux plantarflexion and abduction [21]. However, a recent trial of older people (78% women) found that a progressive, resistance exercise program improved hallux plantarflexion strength by approximately 20% over 12 weeks and resulted in an improvement in perceived foot health [22].

The available evidence provides preliminary support for the use of footwear, foot orthoses and foot exercises for the treatment of hallux valgus, but these three promising approaches are yet to be evaluated in combination. Therefore, the primary objective of this study is to evaluate the feasibility of conducting a randomised trial comparing multifaceted, non-surgical intervention (footwear, foot orthoses, foot exercises, advice, and self-management) versus advice and self-management alone for reducing pain associated with hallux valgus. The secondary objective is to obtain statistical parameters to inform the main trial sample size calculation and provide a signal of efficacy to justify the future main trial.

## Methods

### Study design

The multifaceted inte﻿rve﻿ntion for hallux valgus (MARVELL) trial will be a parallel group, participant- and assessor-blinded, randomised pilot and feasibility trial over 12 weeks. We consider the trial to be both a *pilot* trial (as it includes the key features of a full randomised trial but on a smaller scale) and a *feasibility* trial (as it addresses questions regarding whether a full trial could be feasibly undertaken) [23]. The study has been registered with the Australian and New Zealand Clinical Trial Registry (ACTRN12621000645853), and was developed in consultation with the Standard Protocol Items: Recommendations for Interventional Trials (SPIRIT) 2013 statement [24] and the CONSORT 2010 statement extension to randomised pilot and feasibility trials [25]. The SPIRIT checklist is provided in the Supplementary file, supplemented by items from CONSORT, as recommended by Thabane and Lancaster [26]. Ethical approval was obtained from the La Trobe University Human Ethics Committee (reference number: HEC20474).

### Participants

Twenty-eight participants will be recruited from the northern suburbs of Melbourne, Victoria, Australia. To be eligible for inclusion, participants must: (i) be aged ≥ 40 years, (ii) be female, (iii) have pain in the big toe joint/s (i.e. first metatarsophalangeal or interphalangeal) for at least 12 weeks, (iv) have big toe joint pain rated at least 3 out of 10 on a numerical rating scale, (v) be able to walk household distances (more than 50 m) without the aid of a walker, crutches or cane, (vi) be capable of understanding the English language in verbal and written form, and (vii) have at least moderate hallux valgus on one or both feet, defined as a score of 2 or more on the validated Manchester scale [27], a tool containing four standardised photographs of varying degrees of hallux valgus deformity. Participants will not be eligible for inclusion if they self-report: (i) surgical treatment for hallux valgus on either foot, (ii) lower limb or partial foot amputation, (iii) an inflammatory rheumatological condition such as gout, rheumatoid arthritis, psoriatic arthritis, axial spondyloarthropathy or connective tissue disease, (iv) a neurological disease which interferes with walking (e.g. Parkinson’s disease), (v) having worn arch-contouring foot orthoses in the past 12 weeks (although flat insoles will be permitted), (vi) performing foot exercises (stretching, mobilisation or strengthening) in the past 12 weeks, or (vii) an injury of lower limb(s) or back that may interfere with reaching their feet. We specifically focused on females in this study due to their higher prevalence of hallux valgus [1] and use of constrictive footwear [28].

### Sample size

This is a pilot and feasibility trial, so it is not fully powered to detect statistically significant differences between the groups. The recommended sample size for feasibility and pilot studies is 12 people per group [29], however to allow for a 15% drop-out rate, we will recruit 28 participants. This is similar to the median sample size of 30 for pilot trials documented in the United Kingdom Clinical Research Network database [30].

### Recruitment and screening

Participants will be recruited using the following methods: (i) postal invitation using a database of patients who receive podiatry treatment at the La Trobe University Health Sciences Clinic, (ii) email distribution to staff members in the School of Allied Health, Human Services and Sport at La Trobe University, (iii) Facebook advertising and (iv) posters placed in the local community. Potential participants will be asked to contact the chief investigator (HBM) to express their interest and will then be screened for eligibility by telephone and/or email by two members of the research team (HBM and PQL).

### Baseline assessments

Participant characteristics will be collected by structured interview at the baseline assessment (approximate duration of two hours) and will include age, height, weight, country of birth, education level, major medical conditions, and medications. The following questionnaires and clinical assessments will also be conducted:

(i) the Manchester scale [27] for hallux valgus;
(ii) foot pain characteristics, including duration and location, using a standardised foot diagram [31];
(iii) history of hallux valgus, including age of onset, family history and previous treatments;
(iv) shoe-wearing history, using a standardised set of images depicting toe-box shapes and heel heights [16];
(v) foot structure, using a 3D laser scanner (INFOOT, I-Ware Laboratory, Japan);
(vi) the Incidental and Planned Activity Questionnaire [32];
(vii) the Credibility/Expectancy Questionnaire [33], which assesses participants’ beliefs about the logic underpinning the intervention and perceptions of how much they may benefit. The CEQ will be administered after randomisation and allocation. The CEQ consists of six items; three are related to credibility and three are related to expectancy. For each item, participants will be asked to rate the credibility of the intervention and their expectations on a 9-point Likert scale. High scores on the scale indicate that the participant considers the intervention to be credible and expects it to be effective.

### Randomisation

Permuted block randomisation will be used to randomise participants on a 1:1 ratio to the control or intervention group using an online randomisation service (www.sealedenvelope.com).

### Study procedure

**Fig. 1:**
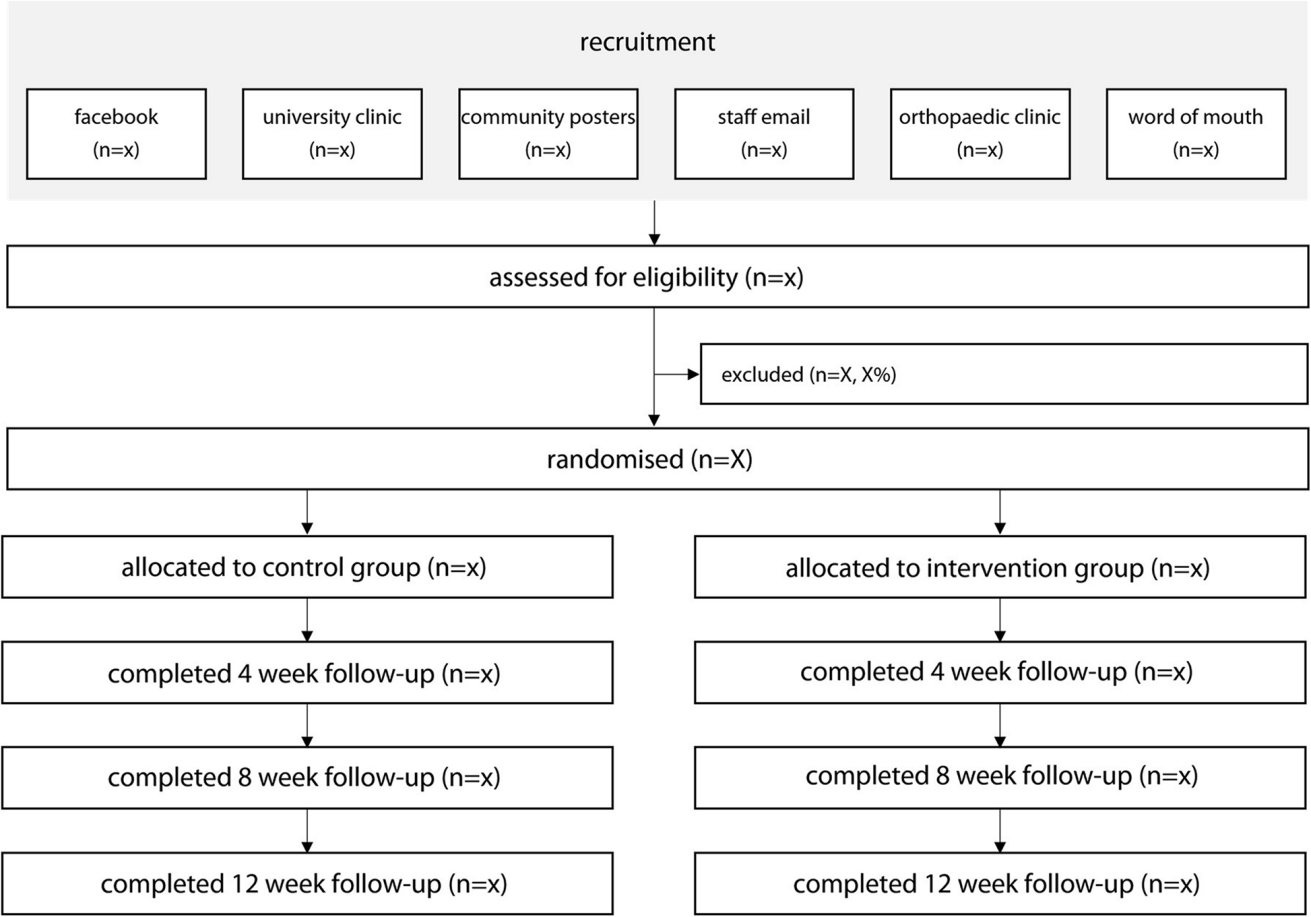
Planned participant flow through the trial

Participant flow through the trial is outlined in Fig. 1. All face-to-face assessments will be performed in the Foot and Ankle Laboratory at La Trobe University, Melbourne, Victoria, Australia. Postal follow-ups will be conducted at 4 and 8 weeks, with the final face-to-face follow-up at 12 weeks.

### Blinding

Participants will be blinded to group allocation by limited disclosure, in that they will be told that the clinical trial is comparing two non-surgical treatments for hallux valgus, but they are not informed about the specific characteristics of the treatments. Research staff administering the treatments cannot be blinded. Outcomes are participant-reported, thus this study is also assessor-blinded (as participants are blinded). The study biostatistician performing the statistical analyses will be blinded.

### Interventions﻿

#### Control group

The control group will receive a self-management package based on United Kingdom National Health Service recommendations [34] which advise people with hallux valgus to wear wide shoes with a low heel and soft sole, apply cold-packs and silicone gel bunion pads, and use paracetamol for pain relief. To address expectations to be provided with a ‘take-home’ intervention, we will provide all participants with cold-packs (Hot + Cold Therapy Gel Pack; OAPL, Clayton, Victoria, Australia) and silicone gel bunion pads (Spandex Gel™ Cushion Bunion Pads; Neat® Feat, Auckland, New Zealand). To meet ethical guidelines and aid retention, on completion of the study the control group participants will be offered the same treatment as the intervention group.

#### Intervention group

**Fig. 2:**
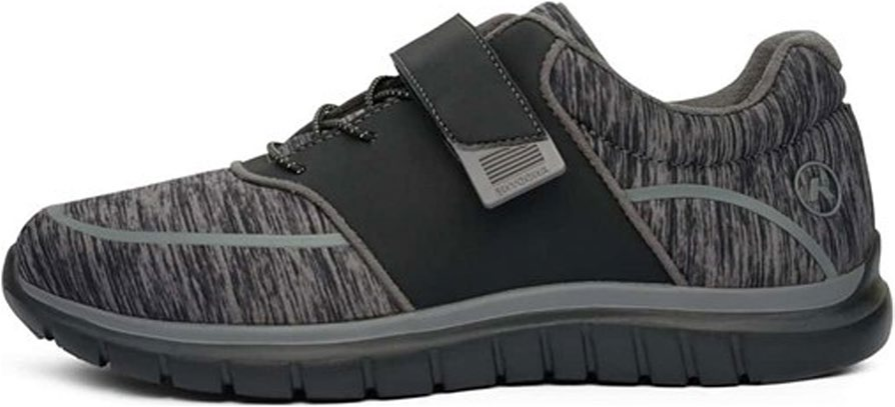
Intervention footwear (Anodyne #45 Sport Jogger). Image reproduced with permission from Global Footcare, Coomera, Queensland, Australia

**Fig. 3:**
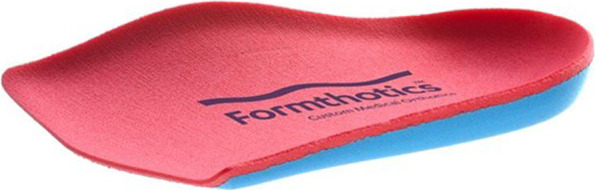
Intervention foot orthoses (dual-density, three-quarter length Formthotics™). Image reproduced with permission from Foot Science International, Christchurch, New Zealand

The intervention group will be provided with the same advice and self-management package as the control group, in addition to:

(i) Footwear: high quality, off-the-shelf footwear (Anodyne #45 Sport Jogger; Global Footcare, Coomera, Queensland, Australia). These shoes have been selected as they have an extra-wide toe box and pliable upper material to alleviate pressure on the hallux (Fig. 2). The shoes will be correctly fitted by a research podiatrist, and participants will be asked to wear them as often as possible.
(ii) Foot orthoses: prefabricated Formthotics™ (Foot Science International, Christchurch, New Zealand). These orthoses are ¾ length and are constructed from dual-density, closed-cell polyethylene foam (bottom layer 140 kg/m^3^, top layer 60 kg/m^3^). See Fig. 3. Previous research has shown that these orthoses are well accepted, with 81% of participants reporting being ‘somewhat or very satisfied’ with them in a 12 month falls prevention trial [35]. We will use the ¾ length rather than full-length devices as they are less likely to increase dorsal/medial pressure from footwear in people with hallux valgus [36]. The existing insoles will be removed from the shoe to accommodate the orthoses. Participants will be asked to wear these with their footwear as often as possible. At the baseline appointment, minor modifications will be made where necessary to ensure that the orthoses are comfortable.
(iii) Foot exercises: participants will be provided with access to a smart-phone app (PhysiTrack®, London, United Kingdom) which demonstrates a home-based version of the progressive resistance foot exercise program developed by Mickle et al. [22]. Participants without access to a smart-phone or personal computer will be provided with a hard copy of the program. The set of nine exercises are performed three times per week for the 12 weeks. Each session takes approximately 30 min to complete. Most of the exercises are performed in a seated position using latex resistance bands wrapped around the toes and/or foot. The exercises target the muscles responsible for ankle dorsiflexion, ankle inversion/eversion, hallux and lesser toe flexion and hallux abduction, and are progressed by either increasing the number of times the exercises are performed (repetitions) or by increasing the level of resistance provided by the bands (light to extra heavy). Adherence to the home-based foot exercise program has been shown to be very high, with 80% of participants (aged 60 to 90 years, 78% women) completing at least 75% of the exercises. The exercises have been shown to increase strength of foot muscles by approximately 20% [22]. Participants will be contacted by the developer of the exercise program (KJM) to address any queries and ensure they are performing the exercises correctly.

Interventions will be administered to both feet, irrespective of whether hallux valgus is unilateral or bilateral. Participants will be free to use additional treatments during the study provided that they are documented in the four-weekly postal surveys. However, participants will be required to withdraw from the study if they report undergoing surgical intervention.

#### Biomechanical assessment

Following randomisation, participants will undergo a biomechanical assessment to evaluate the immediate effects of the footwear/orthotic intervention on loading (i) beneath the foot, and (ii) on the medial aspect of the first metatarsophalangeal joint and hallux. To assess loading beneath the foot, peak plantar pressures under the toes, forefoot, midfoot and heel will be measured with the in-shoe pedar®-X system (novel GmbH, Munich, Germany), a reliable, valid and accurate measure of in-shoe pressure [37]. The pedar® insoles are approximately 2 mm thick and consist of 99 capacitive pressure sensors, arranged in grid alignment. Plantar pressure data will be sampled at a frequency of 50 Hz. Participants will complete four walking trials for each condition, (i.e., own shoes for control group participants and own shoes *versus* extra-depth shoes with orthoses for intervention group participants). An average recording will be determined from the 16 steps (four steps from four trials) for each condition, which has been shown to be a sufficient number of trials to obtain reliable measurements [37].

To assess loading on the medial aspect of the first metatarsophalangeal joint and interphalangeal joint of the hallux, the pedar®-pad, a highly elastic strap of 256 sensors, will be placed inside the shoe along the medial border of the forefoot (incorporating the first metatarsophalangeal and interphalangeal joints), and peak pressures will be documented. This will be performed in participants’ own most frequently worn closed-in shoes (for control group participants) and own shoes *versus* extra-depth shoes with orthoses (for intervention group participants).

### Primary outcome: feasibility

The primary outcome is feasibility, which will be evaluated according to demand, acceptability, adherence, adverse events and retention rate [38]. The measures and thresholds required to demonstrate feasibility are described below, and a summary is provided in Table 1.

**Table 1:** Summary of feasibility outcome measures and thresholds

	*Measure*	*Threshold*
Demand	Recruitment rate	6 participants per month
	Conversion rate	≥ 75%
Acceptability	MOS questionnaire	≥ 75% of the intervention group score more than 5/10 for each of questions 1–6
Adherence		
Footwear/orthoses	Orthotimer® sensor 4-weekly diaries	≥ 75% of participants wear the footwear/orthoses for an average of ≥ 5 h per day
Exercise	PhysiTrack® app / diary	≥ 75% of participants complete at least 24/36 (66%) of the exercise sessions
Adverse events	four-weekly diaries	< 15% and no serious events
Retention rate	Proportion of participants followed up at 12 weeks	≥ 80% retention

#### Demand

Demand will be determined by the recruitment rate (the number of participants recruited per month) and the conversion rate (the proportion of participants providing consent of those who met the selection criteria). The recruitment rate will be considered acceptable if six eligible participants are recruited per month, and the conversion rate will be considered acceptable if ≥ 75%.

#### Acceptability

Acceptability of the intervention will be determined using questions from the Monitor Orthopaedic Shoes (MOS) questionnaire [39] which address issues such as appearance, comfort, weight, and ease of donning and doffing. The intervention will be considered acceptable if ≥ 75% of the intervention group score more than 5/10 for each of questions 1–6.

#### Adherence

Adherence to the footwear/orthoses intervention will be documented using four-weekly diaries and objectively assessed over 12 weeks using a small (9 × 13 × 4.5 mm) temperature sensor embedded in the orthosis (Orthotimer®, Balingen, Germany). Time, date, and temperature measurements will be stored every 15 min, with recordings above 26° Celsius being indicative of shoe wear time [40]. The sensor has previously been validated against objectively measured wear time [40, 41] and is not influenced by ambient temperature fluctuations or physical activity levels [40]. Adherence will be considered acceptable if ≥ 75% of participants wear the footwear/orthoses for an average of ≥ 5 h per day over the 12-week follow-up period. Adherence to the exercise program will be documented using 4-weekly diaries (or the PhysiTrack® smart-phone app) and will be considered acceptable if ≥ 75% of participants attempt at least 66% of the total number of exercise sessions (i.e., 24 out of 36 sessions). In both the control and intervention groups, adherence to the hot/cold packs and bunion pads will be measured using four-weekly diaries.

#### Adverse events

Adverse events will be assessed at four-weekly intervals via postal diary. Participants will be asked to document the type of adverse event, the body location, the frequency and/or severity of the effect. An independent assessor will assess all adverse events as *unrelated*, *probably related* or *definitely related*, and only those considered to be *probably* or *definitely* related will be considered an adverse event. Serious adverse events will be defined as events that are life-threatening, require hospitalisation, or result in persistent or significant disability or incapacity [42]. The rate of adverse events will be considered acceptable if < 15% and none are considered serious.

#### Retention rate

Retention rate is the proportion of recruited participants who complete the 12-week outcome assessment. A ≥ 80% retention rate in each group will be considered acceptable.

### Secondary outcome: limited efficacy testing

The selection of efficacy outcome measures for this trial is based on expert (OMERACT: Outcome Measures in Rheumatology Initiative) guidelines. The key outcome measure is the pain subscale of the Manchester-Oxford Foot Questionnaire (MOXFQ) [43], which has undergone extensive psychometric validation and is recognised as the best condition-specific outcome measure for hallux valgus [44]. The MOXFQ consists of 16 items reflecting three subscales (pain, walking/standing and social interaction). The pain subscale consists of five items scored on a five-point Likert scale (0 to 4; ‘4’ assigned ‘most severe’; higher scores denoting higher severity). The score for each subscale is calculated as the sum of each individual item score and is expressed on a metric of 0 to 100 (100 times actual score, divided by the maximum possible domain score). The MOXFQ pain domain will be measured at baseline and at four-weekly intervals. The pre-specified primary endpoint will be the 12-week score. The minimum clinically important difference for the MOXFQ pain subscale is 12 points [45].

Other limited efficacy outcome measures will include:

(i) the MOXFQ [43] walking subscale, measured at baseline and at four-weekly intervals until 12 weeks;
(ii) the MOXFQ [43] social subscale, measured at baseline and at four-weekly intervals until 12 weeks;
(iii) foot and ankle muscle strength, measured with a hand-held dynamometer using our previously documented, reliable protocol at baseline and week 12 [46];
(iv) general health-related quality of life, assessed using Short Form(SF)-12 [47] measured every four weeks;
(v) number of participants using co-interventions, documented every four weeks;
(vi) participants’ perception of overall treatment effect, assessed with the question “Overall, how has your foot pain changed since the start of the study?” and using a global impression of change 15-point Likert scale response (ranging from ‘a very great deal worse’ to ‘a very great deal better’), measured at 12 weeks [48].

An acceptable feasibility outcome for the limited efficacy testing will be a signal of efficacy for each continuously-scored outcome measure, as evidenced by at least a small effect size (Cohen’s *d* ≥ 0.20, calculated as the difference between the two group means divided by the overall standard deviation), less than 20% use of cointerventions, and a greater than 25% difference in proportion of participants reporting at least ‘somewhat better’ on the perception of overall treatment effect compared to the control group.

A summary of the limited efficacy outcome measures is provided in Table 2, and a summary of standard protocol items is provided in Table 3.

**Table 2:** Summary of the limited efficacy outcome measures

*Measure*	*Threshold*
MOXFQ subscales	small effect size (*d* ≥ 0.20)
Muscle strength	small effect size (≥ 0.20)
SF-12	small effect size (*d* ≥ 0.20)
Use of cointerventions	< 20%
Perception of overall treatment effect	≥ 25% difference in proportion of participants reporting at least “somewhat better” compared to control group

**Table 3:** Summary of standard protocol items

	T0	T1 Baseline (lab)	T2 4 weeks (postal)	T3 8 weeks (postal)	T4 12 weeks (lab)
Postal invitation	X				
Telephone screening	X				
Informed consent		X			
Baseline assessments					
Medical history questionnaires		X			
3D foot scanning		X			X
Biomechanical assessment		X			
Randomisation		X			
Receive intervention		X			
CEQ		X			
MOS (intervention group only)		X			X
Outcome measures					
MOXFQ		X	X	X	X
Muscle strength		X			X
SF-12		X	X	X	X
Use of cointerventions		X	X	X	X
Incidental and planned activity questionnaire		X	X	X	X
Perception of overall treatment effect			X	X	X
Adherence					
Footwear/orthoses		continuous (Orthotimer®)			
Exercise		continuous (PhysiTrack® app)			
Adverse events		X	X	X	X

### Data management

Hard copy baseline and follow-up questionnaires will be stored in a locked filing cabinet at La Trobe University and then shredded after being transferred into electronic formats for analysis (Microsoft Excel; Microsoft Corporation, Redmond, Washington, USA, and IBM SPSS Statistics; IBM Corporation, Armonk, New York, USA). Other data files (3D foot scans and plantar pressure files) will be stored in native file formats on a password-protected university server and will be securely deleted following de-identification and transfer to electronic formats for analysis.

### Statistical analysis

As this is a feasibility study, it is not powered to detect changes in outcome measures, so the focus will not be on inferential testing. Descriptive statistics will be used to report feasibility outcomes. Mean (SD) scores and mean differences (95% CI) will be used to explore differences in continuous variables between the groups. Differences in the MOXFQ pain subscale between groups at 12 weeks (analysis of covariance, adjusted for baseline differences) will be used to inform the sample size calculation for the main randomised trial.

## Discussion

The primary objective of this study is to evaluate the feasibility of conducting a randomised trial comparing multifaceted, non-surgical intervention (footwear, foot orthoses, foot exercises, advice, and self-management) versus advice and self-management alone for reducing pain associated with hallux valgus. Although our recent systematic review [15] identified two studies reporting significant reductions in pain with the use of foot orthoses [19] and exercises [49] in people with hallux valgus, to the best of our knowledge, this is the first study designed to evaluate the effectiveness of these interventions when used in combination, which is more reflective of contemporary clinical practice [14].

A key limitation of the study design is the inability to blind the research staff administering the interventions, however as the outcomes are participant-reported and participants are blinded by limited disclosure, the study can be considered to be assessor-blinded. The most likely barrier to acceptability of (and therefore adherence to) the intervention is aesthetic concerns regarding the footwear. This is a common and largely unavoidable issue with footwear interventions, given their unique role as both a treatment and an item of clothing [50]. Acceptability may be of particular concern in this study, due to the shoes requiring an extra wide toe box to alleviate pressure from the deformity.

The study design has several key strengths, including randomisation, concealed allocation, and blinded analysis, and the reported protocol adheres to the SPIRIT 2013 statement [24] and the CONSORT 2010 statement extension to randomised pilot and feasibility trials [25]. We have selected intervention components that have demonstrated safety and acceptability, are relatively low cost and accessible in the Australian context, so the package of interventions is likely to be easily implemented and scalable if feasibility is demonstrated and the subsequent fully-powered trial demonstrates clinical efficacy. The study design also incorporates objective measurement of adherence, limited efficacy outcome measures addressing the relevant domains of pain and muscle strength, and plantar pressure assessment to provide insights into the potential mechanisms by which footwear and foot orthoses may alleviate loading on the first metatarsophalangeal joint.

### Trial status

The study commenced recruitment in June 2021 and the first participant completed baseline testing on July 8, 2021. However, due to stay-at-home restrictions resulting from the COVID-19 pandemic, recruitment and data collection were suspended from July 15 to 27 and from August 5 to October 21, 2021 (94 days in total) [51]. The study recommenced on October 22, 2021, and we envisage that 12-week follow ups will be completed by July 30, 2022. The findings will be reported to participants, disseminated via peer-reviewed journal articles and presented at international conferences in 2022/3.

## Supplementary Information

**Additional file 1.**

## Data Availability

On completion and publication of the study, de-identified data may be accessed by request from the corresponding author.

## Abbreviations

CEQ: Credibility/Expectancy Questionnaire
CONSORT: Consolidated Standards of Reporting Trials
GP: General Practitioner
MOS: Monitor Orthopaedic Shoes
MOXFQ: Manchester-Oxford Foot Questionnaire
NICE: National Institute for Health Care Excellence
OMERACT: Outcome Measures in Rheumatology
SF: Short Form
SPIRIT: Standard Protocol Items: Recommendations for Interventional Trials
